# Is There a Therapeutic Window for Metabolism-Based Cancer Therapies?

**DOI:** 10.3389/fendo.2017.00150

**Published:** 2017-07-04

**Authors:** Sarah-Maria Fendt

**Affiliations:** ^1^Laboratory of Cellular Metabolism and Metabolic Regulation, VIB Center for Cancer Biology, VIB, Leuven, Belgium; ^2^Laboratory of Cellular Metabolism and Metabolic Regulation, Department of Oncology, KU Leuven and Leuven Cancer Institute (LKI), Leuven, Belgium

**Keywords:** metabolism, cancer, treatment, metastasis formation, endothelial cells, immune cells, cancer cells

Cells that have undergone an oncogenic transformation have an altered metabolism compared to the cells they originate from (1). This observation led to the addition of “a deregulated metabolism” to the hallmarks of cancer (2). Accordingly, it has been extensively demonstrated that many of the observed alterations in the metabolism of cancer cells are important for their proliferation (1, 3, 4). However, a metabolic alteration that is important for cancer cell proliferation is not automatically a good target for treatment, as treatments also have to be selective toward cancer cells. Since almost all of the cancer-induced metabolic changes are not caused by gain of function mutations in specific enzymes, metabolism-based drug have to be developed against the naturally occurring enzymes. Thus, the valid question arises whether there is a therapeutic window for targeting the deregulated metabolism of cancer cells. In the following, I would like to describe the challenges and advocate the opportunities for metabolic drug targets in cancer treatment. In the first section, I will address the question whether there is in general a therapeutic window for metabolism-based cancer treatment, while in the second section, I will discuss new concepts that can refine metabolism-based anticancer strategies.

## Therapeutic Window

Is there a therapeutic window for metabolism-based cancer treatment? A major challenge for metabolic drugs in cancer treatment is that metabolism is a universal cellular process and, with a few exceptions (such as gain of function mutations in metabolic enzymes), the metabolic alterations found in cancer cells are present in similar form in some non-transformed cell; i.e., while cells that undergo an oncogenic transformation will always change their metabolism, there is no single metabolic change that unifies all cancer cells and separates them from all non-transformed cells. Based on this fact, one could argue that targeting the metabolism of cancer cells is challenging, since it is not selective. However, an opportunity for treatment arises based on the fact that many metabolic changes in cancer cells support cell proliferation, while the majority of the non-transformed cells are in a differentiated and low proliferative state. Thus, metabolic drugs that impair cellular proliferation preferentially target cancer cells. The validity of this reasoning is supported by the fact that many of the first chemotherapeutic agents that are still used in the clinics are targeting the metabolism of proliferating cancer cells (5). Examples are the antifolate methotrexate and the nucleoside analog 5-fluorouracil. Despite the fact that these agents target any highly proliferating cell rather than only cancer cells, their usage has revolutionized cancer treatment and the benefits still justify the side effects arising from their moderate selectivity. Thus, metabolism-based treatments are feasible, currently used in the clinics, and a patient benefit at least in the scope of a typical standard of care chemotherapeutic agent can be expected.

Yet, is it possible to refine metabolism-based cancer therapies by increasing efficacy and selectivity and thus broaden the treatment window that arises from the metabolic changes that occur in cancers? In the following, I will focus on three concepts that aim to refine metabolism-based anticancer drugs.

## Metabolic Vulnerabilities Arising from the Cancer-Specific Genetic Landscape

One of the earliest approaches to refine metabolism-based anticancer drugs has focused on metabolic vulnerabilities that arise due to the genetic loss of tumor suppressors or hyperactivation of oncogenes. The rationale for this approach is that many tumor suppressors and oncogenes regulate metabolic genes and consequently loss or hyperactivation of this regulation creates dependencies on specific metabolic pathways (6). This approach led to the identification of an oncogene specific and targetable metabolism in cultured cancer cells. Yet, recent *in vivo* data show that the organ microenvironment and the cell origin can redefine the oncogene-imposed metabolic dependencies of cancer cells and thus can lead to impaired *in vivo* efficacy of metabolic drugs (7–11). A solution to this challenge is the integration of oncogene profiles with the cell origin and the organ microenvironment. An example for the validity of this concept is the finding that cancers with Kras^G12D/+^; Trp53^−/−^ background originating and growing in the lung are susceptible to branched chain amino acid metabolism inhibition, while this is not the case for cancers with the same genetic background but originating and growing in the pancreas (10). Thus, the cancer-specific oncogene and tumor suppressor landscape can be exploited to increase the efficacy of metabolic drugs in the context of the cell origin and the organ microenvironment.

**Figure 1:**
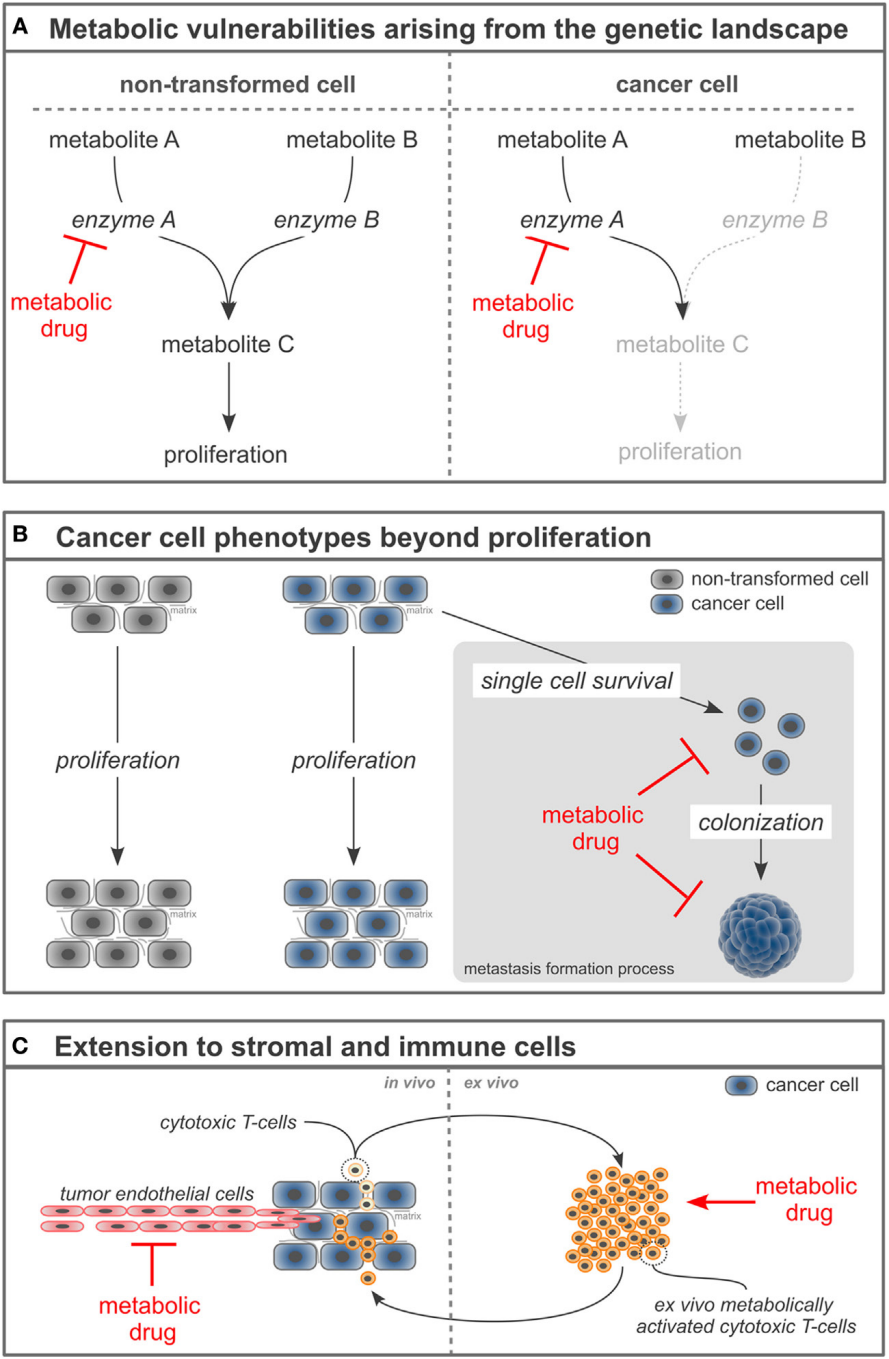
Novel concepts to refine metabolism-based cancer therapies. **(A)** Loss or mutation of enzymes in cancer cells can create hypersensitivity of the cancer cells toward the inhibition of a concomitant enzyme. **(B)** Targeting cancer cell phenotypes beyond proliferation such as single cell survival and colonization can increase the selectivity of metabolism-based drugs and broaden their application toward metastases prevention and treatment. **(C)** Manipulating the cellular tumor composition by targeting stromal and immune cells with metabolism-based drugs can enable a comprehensive cancer therapy.

Another concept that builds on the genetic landscape of cancers to increase the selectivity of metabolic drugs focuses on the metabolic vulnerabilities arising from a mutation in or gene loss of a metabolic enzyme (Figure 1A). The rationale of this concept is that normal cells have the metabolic flexibility to cope with drugs that (partially) inhibit an enzyme, while cancer cells fail to have this flexibility due to a mutation or loss in an enzyme concomitant to the enzyme targeted by the drug. An example for this concept are cancers with homozygous loss of p16/CDKN2A resulting in the passenger deletion of the enzyme methylthioadenosine phosphorylase (MTAP) (which is found in ~15% of all cancers and>50% of glioblastoma multiforme) and inhibition of the enzyme arginine methyltransferase (PRMT5) (12–14). Mechanistically, loss of MTAP results in the accumulation of its metabolite substrate methylthioadenosine, which partially inhibits PRMT5 activity. Consequently, cancers with loss of MTAP and therefore already impaired PRMT5 activity are hypersensitive toward PRMT5 inhibitors (12–14). Another example for this concept is demonstrated by the effectiveness of a pyruvate carboxylase (PC) knockdown to impair the proliferation of paraganglioma with mutation in succinate dehydrogenase (SDH) (15, 16). Mechanistically, mutations in SDH result in a truncated tricarboxylic acid cycle and therefore impaired glutamine anaplerosis (17), which is a process that supports aspartate production required for nucleotide biosynthesis. Consequently, SDH mutant tumors switch to PC-dependent anaplerosis to sustain nucleotide biosynthesis. In turn, SDH mutant tumors are hypersensitive toward PC knockdown, while non-transformed cells have the flexibility to use either path of tricarboxylic acid cycle anaplerosis. Thus, combining the genetic loss of an enzyme with a metabolic drug creates hypersensitivity specifically in cancer cells. Taken together, identifying the metabolic vulnerabilities that arise from the cancer-specific genetic landscape can be conceptualized to increase the selectivity and efficacy of metabolic drugs.

## Cancer Cell Phenotypes Beyond Proliferation

A recent concept to refine metabolic drugs is focused on understanding the metabolic vulnerabilities of metastasizing rather than proliferating cancer cells. As described in the first section, most metabolism-based anticancer drugs inhibit the proliferation of cancer cells (1, 18). Unquestionable, this is a very important aspect of cancer therapy. However, this focus on proliferation contributes to the moderate selectivity of metabolism-based drugs (and many other drugs that target cancer cell proliferation), since some non-transformed cells also proliferate. A solution to this challenge is the concept to go beyond the proliferative phenotype of cancer cells and target their single cell survival and colonization capacity (Figure 1B). These latter phenotypes are less frequently found in normal cells compared to the proliferation phenotype. Moreover, they are particularly important for cancer progression toward metastasis formation, which results in up to 90% of the patient mortality. Thus, considering phenotypes beyond proliferation can increase selectivity of metabolic drugs and advance their application toward inhibition of metastasis formation. An example for this concept is the recent discovery that inhibition of proline catabolism impairs metastasis formation by breast cancer cells without apparent adverse effects on normal cells and organ function (19). Mechanistically, metastasizing cancer cells rely on proline catabolism to fuel their increased energy need during the colonization of distant organs. Consequently, targeting proline metabolism does not affect primary cancer growth or non-transformed cells, but impairs metastasis formation in distant organs (19). Another example for this concept is the finding that the survival of metastasizing cancer cells in the circulation depends on their antioxidants metabolism (20). Consequently, targeting one carbon metabolism that contributes *via* NADPH production to the cellular antioxidants response [e.g., by inhibiting methylenetetrahydrofolate dehydrogenase (MTHFD1)] decreases the survival of cancer cells in the circulation and subsequently metastasis formation in distant organs (20). Taken together, targeting cancer cell phenotypes beyond proliferation refines metabolic drugs and extends their application toward anti-metastatic agents.

## Extension to Stromal and Immune Cells

An additional concept to refine the use of metabolic drugs in cancer treatment is targeting the entire cellular composition of a cancer, which includes stromal and immune cells. Classically, metabolism-based drugs have been developed against cancer cells. However, within the tumor, not only cancer cells but also stromal and immune cells are found. Many stromal and some immune cells (such as tumor-associated macrophages) are reprogrammed to support the development and progression of cancer, while other immune cells within the tumor (such as cytotoxic T-cells) counteract cancer development and progression. Thus, targeting stromal and/or immune cells along with the cancer cells can be a comprehensive treatment concept (Figure 1C). The effectiveness of this concept has been shown for stromal cells: tumor endothelial cells display an aberrant activation (in form of proliferation and migration), which leads to tumor vascularization, but also vascular permeability. This aberrant activation is at least in part driven by high glycolytic rates (21). Consequently, downregulating glycolysis in tumor endothelial cells can normalize the tumor vasculature, which has been shown to result in increased efficacy of chemotherapy and decreased metastasis formation (21). Both effects relied on a tightened vascular barrier that resulted in improved delivery of chemotherapeutic agents to the cancer and decreased success of cancer cell intravasation to the vasculature. Thus, targeting tumor-associated stromal cells and cancer cells at the same time can provide a synergistic anticancer efficacy.

Targeting the metabolism of immune cells emerges to be more complex, since the different subclasses of immune cells exhibit either pro- or antitumor capacities (22, 23). Therefore, any metabolism-based therapy targeting immune cells needs to either hamper the fitness of immune cells with protumor capacity or boost the fitness of immune cells with antitumor capacity. To achieve such selectivity, an increased understanding of the metabolism of immune cells is needed. An approach to circumvent the above-described complexity is to stimulate the metabolic fitness of antitumor immune cells *ex vivo* and combine it with a consecutive adoptive transfer. For example, it has been shown that the *ex vivo* treatment of cytotoxic T-cells with the metabolite S-2-hydroxyglutarate (not to be confused with the oncometabolite R-2-hydroxyglutarate) results (after adoptive transfer) in enhanced *in vivo* proliferation, survival, and antitumor capacity of the treated cytotoxic T-cells (24). Mechanistically, S-2-hydroxyglutarate treatment induced changes in histone and DNA methylation as well as the activation of HIF-1α-dependent transcriptional programs (24). Thus, while approaches targeting the metabolism of immune cells *in vivo* require further research, *ex vivo* approaches show promising results. Taken together, targeting the metabolism of stromal and immune cells can refine cancer treatment.

In conclusion, metabolism-based drugs are important contributors to cancer treatment. Novel concepts such as targeting metabolic vulnerabilities of cancer cells arising from their genetic landscape, metabolic requirements of metastasizing cancer cells, and stromal and immune cells have the potential to refine metabolism-based anticancer therapies. Moreover, combining current and future metabolism-based drugs with targeted delivery such as nanobodies (25) and magnetic nanoparticles (26) can further advance their use in cancer treatment. Thus, my answer to the question “Is there a therapeutic window for metabolism-based cancer therapies?” is *yes*.

## Author Contributions

The author confirms being the sole contributor of this work and approved it for publication.

## Conflict of Interest Statement

The author declares that the research was conducted in the absence of any commercial or financial relationships that could be construed as a potential conflict of interest.
